# SOX chemotherapy with anti-PD-1 and iNKT cell immunotherapies for stage IV gastric adenocarcinoma with liver metastases: A case report

**DOI:** 10.3389/fimmu.2022.1073094

**Published:** 2022-12-12

**Authors:** Dezhao Li, Mei Liu, Jinhuan Wang, Jia Guo, Ningzhi Xu, Jun Lu

**Affiliations:** ^1^ Hepatology and Cancer Biotherapy Ward, Beijing YouAn Hospital, Capital Medical University, Beijing, China; ^2^ Laboratory of Cell and Molecular Biology, National Cancer Center/National Clinical Research Center for Cancer/Cancer Hospital, Chinese Academy of Medical Sciences and Peking Union Medical College, Beijing, China; ^3^ State Key Laboratory of Molecular Oncology, National Cancer Center/National Clinical Research Center for Cancer/Cancer Hospital, Chinese Academy of Medical Sciences and Peking Union Medical College, Beijing, China

**Keywords:** invariant natural killer T cell, gastric cancer, multimodal therapy, pathological complete response, case report

## Abstract

Gastric cancer (GC) is the fourth most common cancer worldwide, with overall 5-year survival rate of approximate 20%. Although multimodal treatments that combine surgery with chemotherapy and immunotherapy have been shown to improve survival, pathological complete response (pCR) is rare in advanced GC patients with liver metastases. Pre-clinical studies and clinical trials have demonstrated the antitumor efficacy of invariant natural killer T (iNKT) cells in various malignancies, including GC. While multimodal therapy comprised of chemotherapy, anti-programmed cell death-1 (PD-1) therapy, and iNKT cell immunotherapy have not been reported in GC patients. This case report describes the treatment of an early 60s patient diagnosed with advanced stage IVB (T1N1M1) adenocarcinomas of gastric cardia with liver metastases who received multimodal therapy comprised of SOX chemotherapy, anti-programmed cell death-1 (PD-1) therapy, and iNKT cell immunotherapy followed by surgical resection. Dramatic decreases in tumor area were observed in both the primary tumor and metastatic lesions following six cycles of SOX chemotherapy and iNKT cell immunotherapy, and four cycles of anti-PD-1 therapy. This combined treatment resulted in the transformation of a remarkably large, unresectable liver metastases into a resectable tumor, and the patient received total gastrectomy with D2 lymph node dissection and liver metastasectomy. Subsequent pathological examination detected no cancer cells in either the primary site or liver metastatic lesions, supporting the likelihood that this treatment achieved pCR. To our knowledge, this report represents the first case of a metastatic gastric cancer patient displaying pCR after six months of multimodal therapy, thus supporting that a SOX chemotherapy, anti-PD-1 therapy, and iNKT cell immunotherapy combination strategy may be effective for treating, and potentially curing, patients with advanced gastric adenocarcinoma.

## Introduction

Gastric cancer (GC) is the fourth most common type of cancer, and the third leading cause of cancer mortality worldwide (1). Among GC patients, those with advanced unresectable and/or metastatic tumors comprise the largest proportion, with loco-regional lymph nodes, liver, and peritoneum representing the frequent metastasis sites (2).

Despite considerable research efforts, GC remains difficult to treat and has a typically poor prognosis for five-year overall survival according to pathological stage and intervention (surgery only IA, 93.6%; IIA, 81.8%, and IIIA, 54.2%; or with neoadjuvant I, 76.5%; II, 46.3%; III, 18.3%; and IV, 5.7%) (1).

Although multimodal treatments that combine surgery with systemic multi-line chemotherapy are generally quite successful for prolonging patient survival, improvements to complete cure rate have lagged for GC, and GC results in mortality for >70% of patients (1).

Chemotherapy regimens that include the 5-FU analog S-1 plus platinum are commonly used to treat stage IV GC, since it confers moderate survival benefits (3). However, anti-programmed cell death protein 1 (PD-1)/programmed death-ligand 1 (PD-L1) immunotherapies are currently generating encouraging preliminary results in clinical GC trials (either as a monotherapy or in conjunction with chemotherapy) (4). Moreover, a previous study reported that a combination of immune-checkpoint blockade and chemotherapy prolonged the survival time for patients with metastatic GC (5).

So far, numerous pre-clinical studies and clinical trials have confirmed the antitumor efficacy of invariant natural killer T (iNKT) cells against various malignancies (6–8). One study specifically in GC patients defined an increase in peripheral IFN-γ-producing iNKT cell counts as a positive response, and suggested that a deficiency for iNKT cell-mediated antitumor immunity may contribute to GC tumor progression (9).

Here, we report a patient with stage IV gastric adenocarcinoma and liver metastases who achieved pathological complete response (pCR) after receiving a multimodal therapy that included SOX chemotherapy, anti PD-1 therapy, and iNKT cell immunotherapy followed by surgical resection.

## Case presentation

An early 60s patient who has 10 years of drinking history, without a history of hypertension, diabetes, or hepatitis, was admitted to hospital after experiencing one month of upper abdominal distension. The patient denied family history of cancer. One month before admission, an ultrasound examination indicated the presence of a hepatic nodule (about 7-8 cm in diameter) and abdominal CT showed occupied intrahepatic solid lesion. The patient felt abdominal distention only that was not relieved after symptomatic treatment with domperidone orally administered. Physical examination showed that the liver could be touched 3 cm below the ribs and 5 cm below the xiphoid process, with tenderness. The patient’s Eastern Cooperative Oncology Group (ECOG) performance status (PS) score was 1. Routine blood and biochemical tests showed no obvious abnormalities except for elevated CEA (231.0 ng/ml; normal range: 0.0–5.0 ng/mL) and CA199 (126.0 U/ml; normal range: 0.00–37.00 U/mL).

**Figure 1:**
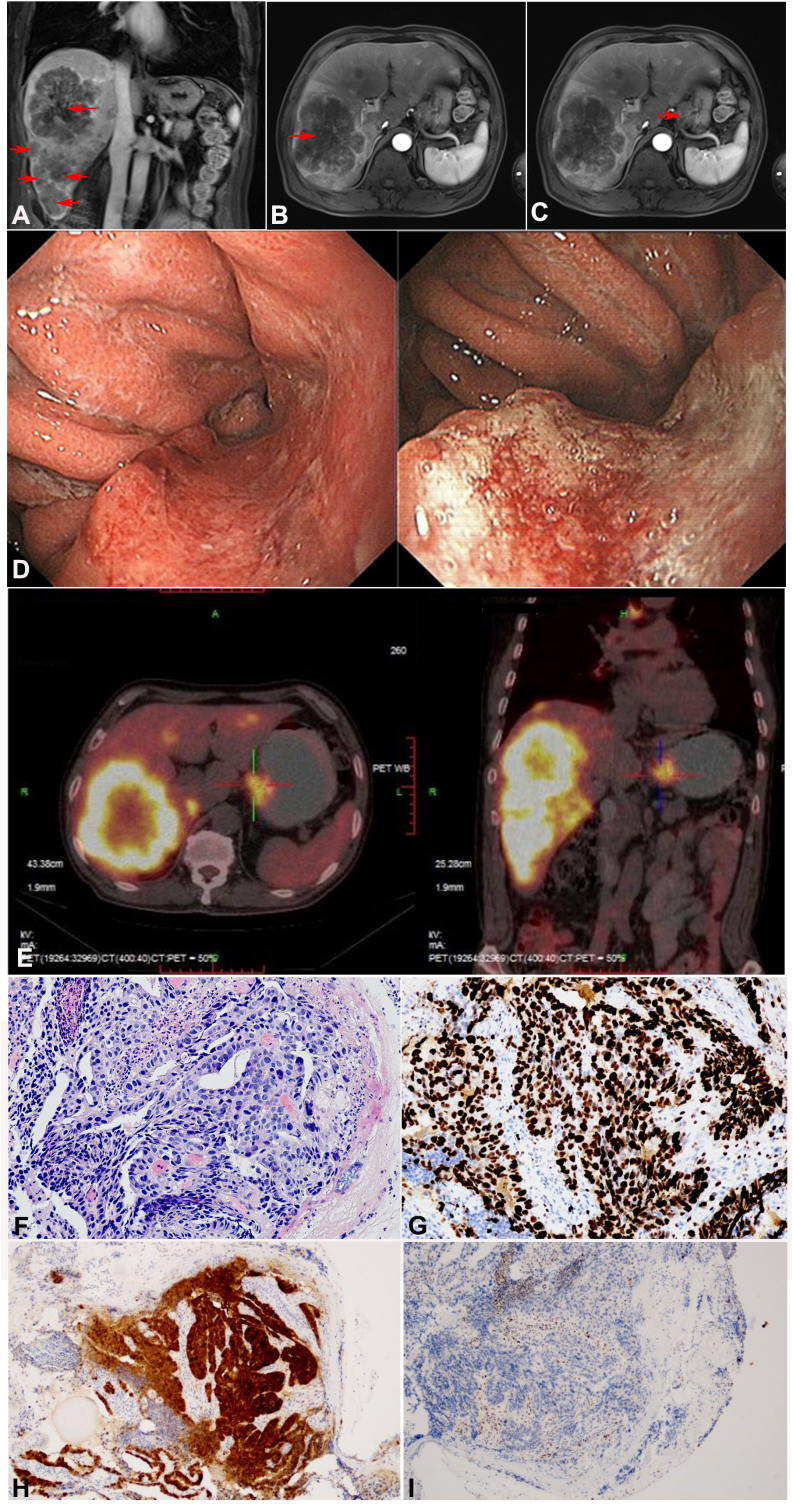
Imaging and pathological examination of the patient before treatment (tumor marked in red arrow). **(A–C)** Abdominal MRI revealed a soft tissue mass in the gastric cardia and multiple scattered nodules in the liver, **(A)** Coronal imaging of the lesion on liver, **(B)** Sagittal imaging of the lesion on liver, **(C)** Sagittal imaging of the lesion on gastric cardia. **(D)** Upper gastrointestinal endoscopy findings before treatment. The posterior wall of the cardia showed surrounding eminence and central ulcerative lesions with an area of 1.8 × 1.8 cm. **(E)** PET-CT showed uneven thickening of the gastric wall in the cardia area, as well as multiple nodules in the liver. **(F–I)** Pathological examination of gastric biopsy specimen confirmed moderately differentiated adenocarcinoma. **(F)** H&E, × 200. **(G)** Ki67, × 200. **(H)** P16, ×100. **(I)** P53, × 100.

Abdominal magnetic resonance imaging (MRI) revealed a soft tissue mass in the gastric cardia and multiple scattered nodules in the liver, with the largest lesion approximately 133 × 106 mm in size (**Figures 1A–C**). Upper gastrointestinal endoscopy showed a bulge around the posterior wall of the cardia and a central ulcerative lesion with an approximate area of 1.8 × 1.8 cm. The posterior wall was uneven, brittle, and bled easily (**Figure 1D**). Positron emission tomography/computed tomography (PET-CT) showed uneven thickening of the gastric wall in the cardia area with mass shadow, as well as multiple nodules in the enlarged liver (**Figure 1E**). Immunohistochemical analysis of gastroscope biopsy specimens were as follows: P53 (-), P16 (+), Ki67 (+ >90%), HP (-), and PD-L1(22C3) (CPS=1) (**Figures 1F–I**).

Based on the above examinations, the patient was clinically diagnosed as stage IVB gastric cardiac adenocarcinoma (GCA) (T1N1M1) with multiple liver metastases, according to criteria in the 8th edition of the AJCC TNM staging system.

The patient received a multimodal therapy comprised of SOX chemotherapy, anti-PD-1 therapy, and iNKT cell immunotherapy followed by surgical resection. The SOX regimen (tegafur S-1 + oxaliplatin) was administered every 21 days. S-1 (40 mg/m^2^, *bid*) was taken orally from day 1 to 14, and oxaliplatin (130 mg/m^2^) was injected intravenously on day 1. Anti-PD-1 mAb (camrelizumab) (200 mg) was administered intravenously once every 21 days. iNKT cells (6~9×10^7^ cells/m^2^ intravenously) were infused twice monthly, followed by subcutaneous injection of interleukin (IL)-2 (25,000 IU/kg) once every other day for 2 weeks. All procedures used to prepare iNKT cells are provided in the supplemental file (**sMethod 1** in **Supplemental File**). Lymphocyte tests and assessments were conducted at baseline and every 4 weeks after iNKT cell infusion until surgery was performed (**sFigure 1** in **Supplemental File**).

**Figure 2:**
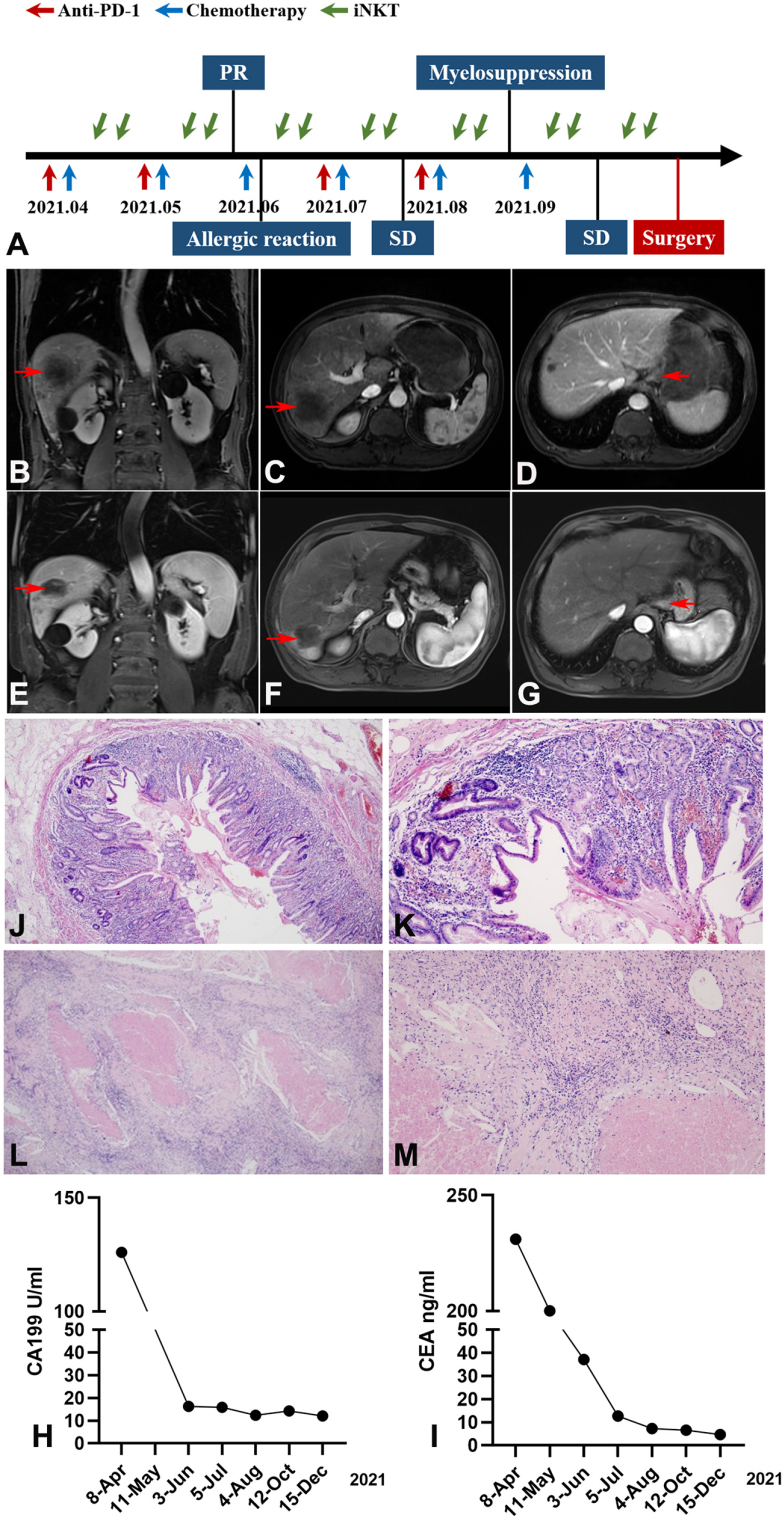
Imaging, pathological and clinical parameters of the patient following study design (tumor marked with red arrow). **(A)** The patient received a multimodal therapy comprised of SOX chemotherapy, anti-PD-1 therapy, and iNKT cell immunotherapy followed by surgical resection. The SOX regimen was administered every 21 days; Anti-PD-1 mAb was administered intravenously once every 21 days; iNKT cells were infused twice monthly. **(B–D)** Abdominal MRI revealed a decrease of the metastatic tumor in the liver and the lesion occupying the cardia region (after 2 cycles of treatment). **(B)** Coronal imaging of the lesion on liver, **(C)** Sagittal imaging of the lesion on liver, **(D)** Sagittal imaging of the lesion on gastric cardia. **(E–G)** Abdominal MRI revealed a further decrease of the largest metastatic tumor in the liver (after 6 cycles of treatment). **(E)** Coronal imaging of the lesion on liver, **(F)** Sagittal imaging of the lesion on liver, **(G)** Sagittal imaging of the lesion on gastric cardia. **(H, I)** The downward trend of CEA and CA-199 during treatment (CEA normal range, 0.0–5.0 ng/mL; CA199 normal range, 0.00–37.00 U/mL). **(J–M)** Pathological examination of gastric and liver biopsy specimen confirmed pCR after surgery. Gastric specimen (**J**, magnification×40, **K**, magnification×100); Liver specimen (**L**, magnification×40, **M**, magnification×100).

**Figure 2A** presents the entire treatment timeline (from April 2021 to September 2021). In June, after two cycles of SOX chemotherapy, anti-PD-1 therapy, and iNKT cell immunotherapy, abdominal MRI revealed that the largest metastatic tumor in the liver had decreased from 133 × 106 mm to 57× 52 mm and that the maximum diameter of the lesion occupying the cardia region had decreased from 36 mm to 20 mm (**Figures 2B–D**).

On the third cycle of SOX chemotherapy, an allergic reaction (rash) occurred after oxaliplatin infusion, and anti-allergic treatment (cetirizine hydrochloride 10 mg + vitamin C 2g + calcium gluconate 20 ml) was given until the rash subsided. Given that dermal toxicity is the most common and one of the earliest immunotherapy-related adverse reactions to immune checkpoint inhibitor (ICI) treatments (10), camrelizumab was not administered at this time.

In August, following the fourth cycle of SOX chemotherapy and iNKT cell immunotherapy, and the third cycle of anti PD-1 therapy, the largest metastatic tumor in the liver decreased from 133 × 106 mm to 51× 47 mm in abdominal MRI, leading to an assessment of stable disease (SD). However, the platelet count was significantly reduced to 36×10^9^/L prior to starting the 6th cycle of SOX chemotherapy, indicating severe myelosuppression. Avatrombopag, recombinant thrombogenietin, and mechanized platelet infusion were orally administered. Considering that ICIs have been associated with severe or even fatal hematologic toxicities-immune thrombocytopenic purpura (ITP) (11), we suspended the anti PD-1 therapy.

After recovery from myelosuppression, oxaliplatin was reduced to 85 mg/m^2^. In October, abdominal MRI of the largest metastatic tumor in the liver showed a further decrease from the original size of 133 × 106 mm to 40 × 37 mm. The tumor was then evaluated as resectable and surgery was scheduled (**Figures 2E–G**). The CEA and CA199 showed a gradual decreasing trend throughout the course of treatment (**Figures 2H, I**).

In November, the patient underwent total gastrectomy with D2 lymph node dissection and liver metastasectomy. Postoperative pathology demonstrated reactive granulomatous lymphadenitis in the lesser curvature of the stomach and coagulative necrosis in the right posterior lobe of the liver, accompanied by granulomatous inflammation; no residual tumors were observed at the surgical margin. Pathological examination found no cancer cells in the primary or liver metastatic lesions, confirming a pCR (**Figures 2J–M**).

The percentage of iNKT cells, NK cells and activated NK cells were higher after the iNKT cell infusion (**sFigure 1** in **Supplemental File**).

After surgery and discharge, the patient continued to have follow-up examinations in our out-patient clinic every three months, including routine blood and biochemical tests, physical examination, imaging examination and the evaluation of quality of life. Patient compliance was good, and at the time this case report was written, the patient had a good quality of life without any signs of recurrence or adverse events.

## Discussion

GC is the fifth most frequently diagnosed cancer and the third leading cause of cancer-related death worldwide. Currently, the only potentially curative treatment approach for patients with gastric cancer is surgical resection with adequate lymphadenectomy, and patients diagnosed with unresectable, locally advanced, or metastatic GC tumors have no available therapeutic options beyond palliative therapies that provide limited extension of survival time (1).

Major advances in recent years include the development of neoadjuvant chemotherapies (NAC), which can increase R0 resection rates by almost 10.0% and inhibit lymph node metastasis (12). However, pCR is rare in advanced GC patients treated with NAC.

ICIs, such as anti-PD-1 mAbs, have become a standard of care for advanced GC, although the response to ICIs remains far from satisfactory. In 2019, the phase III KEYNOTE-062 trial showed that for advanced gastric or gastroesophageal junction (G/GEJ) adenocarcinoma, chemotherapy plus pembrolizumab therapy did not confer a survival benefit as compared with the chemotherapy alone (13). A double-blind phase 3 trial revealed that ramucirumab with cisplatin and fluoropyrimidine as a first-line therapy did not improve overall survival for patients with metastatic gastric or junctional adenocarcinoma (14).

There are currently several strategies in development to increase the clinical efficacy of ICIs. One main goal of these strategies is to restore immune cell populations, thereby “resetting” the immune system from a tolerogenic to an immunogenic state (15).

Invariant NKT is a type of natural killer T cell that can produce large quantities of cytokines, such as the Th1-type cytokine interferon-γ (IFN-γ) and the Th2-type cytokine interleukin-4 (IL-4), to promote dendritic cell maturation, enhance their capacity for antigen presentation, and subsequently reinforce CD8^+^ T cell responses (16). The results of our phase 1 trial indicated that autologous iNKT cell therapy is well-tolerated by hepatocellular carcinoma (HCC) patients (17), suggesting its potential to improve outcomes in other cancers. In addition, elevated PD-1 levels were found to be associated with iNKT cell deficiency (18), indicating that iNKT cells likely contribute to overcoming poor immune cell response in tumors.

A recent study showed that a circulating subset of iNKT cells with NK cell-like properties in the peripheral blood in human exhibit enhancing antitumor immunity and antiviral immune responses (19). Accumulating evidence in numerous human cancers supports a strong positive correlation between overall survival and the frequency and function of iNKT cells in the tumor or in circulation (20–24). The deficiency for iNKT cell-mediated antitumor immunity may contribute to GC tumor progression (9), therefore the increase in peripheral iNKT cells after the infusion of iNKT cells may be considered a positive response and the related experiment is in progress.

In the present case report, the patient chose to try this multimodal therapy comprised of SOX chemotherapy with anti-PD-1 and iNKT cell immunotherapies followed by surgical resection after consulting many medical institutions. With the dramatic decreases in tumor area and the improvement of life quality, the patient became more optimistic with the treatment and achieved pCR.In addition, compared with T cells, iNKT cells are not restricted by MHC but presented by CD1d molecules. Extensive pre-clinical and clinical evidence demonstrate an attractive role of iNKT in protecting from graft-versus-host-disease (GvHD), which supports that iNKT-based immunotherapy could be derived from healthy donor without risk of GvHD. In the future, immunotherapy based on iNKT will be available “on the shelf” and the financial burden will be greatly reduced.

In general, many multimodal treatments combined surgery with chemotherapy and immunotherapy have been shown to improve survival, but pCR is rare in advanced GC patients with liver metastases. The multimodal therapy presented in this report resulted in the transformation of a remarkably large, unresectable liver metastases into a resectable tumor, and displaying pCR in this stage IVB GC patient with liver metastases. This multimodality strategy may thus emerge as a promising option for patients with locally advanced, unresectable, and/or advanced metastatic GC. However, the biomarker for clinical outcome prediction based on the multimodal treatments needs to be further studied, and the monitoring for engraftment of iNKT cell needs to be more meticulous.

## Data availability statement

The original contributions presented in the study are included in the article/**Supplementary Material**. Further inquiries can be directed to the corresponding author.

## Ethics statement

All human sample collection and the usage were approved by the ethics committee of Beijing Youan Hospital (Jing You Ke Lun [2018] No. 016) and were conducted according to the principles of the Declaration of Helsinki. Written informed consent was obtained from the individual(s) for the publication of any potentially identifiable images or data included in this article.

## Author contributions

JL contributed to the project design and supervised the work. DL contributed to the process of patients’ cell infusion, sampling, and data collection, and wrote the manuscript. ML edited the manuscript and drafted figures. JW and JG contributed to relevant data collection, patient management, and sample collection. NX provided technical support. All authors contributed to the article and approved the submitted version.
