# Supplementary material for: SOX chemotherapy with anti-PD-1 and iNKT cell immunotherapies for stage IV gastric adenocarcinoma with liver metastases: A case report
Source: Front Immunol. 2022 Dec 12;13:1073094. doi: 10.3389/fimmu.2022.1073094 (PMC9792132; doi:10.3389/fimmu.2022.1073094)
Supplement: Supplementary file 1 [file DataSheet_1.docx]

**Supplemental Content**

**sFigure 1. Lymphocytes tests and assessments after iNKT cells infusion.**


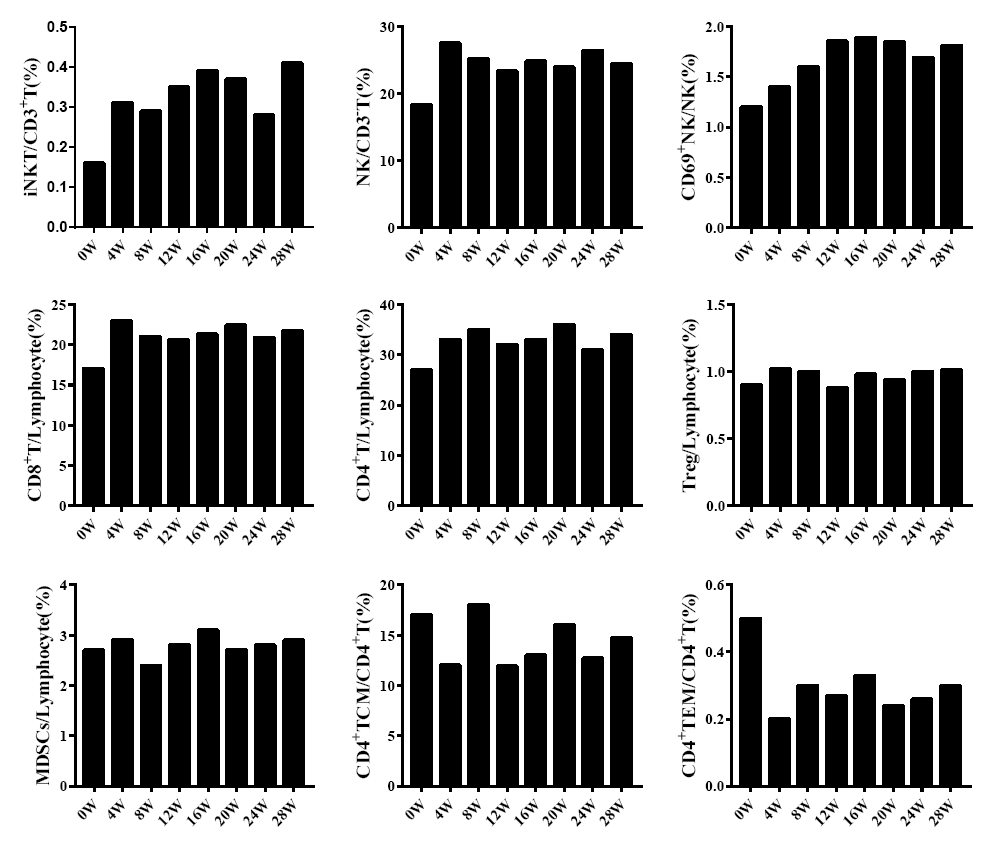


**sMethod 1. Preparation of iNKT Cells.**

1) Isolate PBMCs by leukapheresis using a COM.TEC apheresis system (Fresenius Medical Care, Bad Homburg, Germany) and Lymphoprep density gradient medium (Stemcell Technologies, Cambridge, UK).

2) Resuspend PBMCs in Corning serum-free cell medium KBM581 (Corning Inc., Corning, NY) and stimulate with 100 ng/mL α-GalCer (BioVision, Milpitas, CA) and 100 U/mL animal-free recombinant human interleukin-2 (rhIL-2; BioLegend, San Diego, CA) for 48 hours; the rhIL-2 was replenished every other day.

3) Stimulate PBMCs with 1,000 IU/mL granulocyte-macrophage colony-stimulating factor (BioLegend, San Diego, CA) and IL-4 (BioLegend, San Diego, CA) for 1 week, and obtain mature, monocyte-derived DCs.

4) Sort iNKT cells by using anti-iNKT microbeads (Miltenyi Biotec, Bergisch Gladbach, Germany) according to the manufacturer’s protocol on day 7.

5) Co-culture the sorted iNKT cells with mature DCs for 14 days, and collect cells for infusion on day 21. An aliquot of cells was used for immunophenotyping and functional testing.

6) Determine the frequency of iNKT cells in T cells by staining with anti-CD3-FITC, anti-TCR Vα24-Jα18-PE. (FACSCanto II cytometer, BD Biosciences, San Jose, CA).

7) The criteria for iNKT cell administration include a negative bacterial culture, a negative mycoplasma test result, and an endotoxin level < 0.05 units/mL.
